# Visible Light-Activated Carbon Dots for Inhibiting Biofilm Formation and Inactivating Biofilm-Associated Bacterial Cells

**DOI:** 10.3389/fbioe.2021.786077

**Published:** 2021-11-18

**Authors:** Xiuli Dong, Christopher M. Overton, Yongan Tang, Jasmine P. Darby, Ya-Ping Sun, Liju Yang

**Affiliations:** ^1^ Department of Pharmaceutical Sciences, Biomanufacturing Research Institute and Technology Enterprise (BRITE), North Carolina Central University, Durham, NC, United States; ^2^ Department of Chemistry, Clemson University, Clemson, SC, United States; ^3^ Department of Mathematics and Physics, North Carolina Central University, Durham, NC, United States

**Keywords:** biofilm, carbon dots, inactivation, inhibition, photoactive

## Abstract

This study aimed to address the significant problems of bacterial biofilms found in medical fields and many industries. It explores the potential of classic photoactive carbon dots (CDots), with 2,2′-(ethylenedioxy)bis (ethylamine) (EDA) for dot surface functionalization (thus, EDA-CDots) for their inhibitory effect on *B. subtilis* biofilm formation and the inactivation of *B. subtilis* cells within established biofilm. The EDA-CDots were synthesized by chemical functionalization of selected small carbon nanoparticles with EDA molecules in amidation reactions. The inhibitory efficacy of CDots with visible light against biofilm formation was dependent significantly on the time point when CDots were added; the earlier the CDots were added, the better the inhibitory effect on the biofilm formation. The evaluation of antibacterial action of light-activated EDA-CDots against planktonic *B. subtilis* cells *versus* the cells in biofilm indicate that CDots are highly effective for inactivating planktonic cells but barely inactivate cells in established biofilms. However, when coupling with chelating agents (e.g., EDTA) to target the biofilm architecture by breaking or weakening the EPS protection, much enhanced photoinactivation of biofilm-associated cells by CDots was achieved. The study demonstrates the potential of CDots to prevent the initiation of biofilm formation and to inhibit biofilm growth at an early stage. Strategic combination treatment could enhance the effectiveness of photoinactivation by CDots to biofilm-associated cells.

## Introduction

Bacterial biofilms represent a significant problem in the medical field. Biofilms not only colonize and damage a wide variety of medical implants and devices, but they also cause a majority of bacterial infections in humans (Singh et al., 2018) with an estimated up to 80% of all human bacterial infections being associated with biofilms (Römling and Balsalobre, 2012). Biofilms are also major concerns in many industries from the production of oil and gas to the distribution of safe drinking water (Ikuma et al., 2013) due to problems such as biofilms causing metal corrosion in engineered systems.

Biofilm formation is a complex process in which microorganisms irreversibly attach to and grow on a surface and produce extracellular polymeric substances (EPS) that facilitate the attachment and formation of an extracellular matrix (ECM), resulting in the altered phenotype of the organisms with respect to growth rate and gene transcription (Donlan, 2001). The mechanical networks of EPS in mature biofilms protect the microorganisms; retain water, organic compounds, inorganic ions, and extracellular enzymes; enable redox activity; and facilitate horizontal gene transfer (Flemming and Wingender, 2010; Marvasi et al., 2010; Sretenovic et al., 2017). Such structure and lifestyle of a biofilm afford it strong capability to withstand hostile environmental conditions and make it much more resistant to antibiotics, disinfection, and/or sanitization when compared with their planktonic bacteria counterparts (Mah and O'Toole, 2001; Bridier et al., 2015). For bacterial cells in some biofilms, their increases in antibiotic tolerance could be up to 1,000 times (Rogers et al., 2010). Although many traditional antimicrobial reagents have been used to prevent biofilm formation or to eradicate mature biofilms (Lechevallier et al., 1988; Gilbert et al., 2001; Pitts et al., 2003; Davison et al., 2010), the chemicals mostly require high dosages and are often toxic, creating potentially major issues in environmental and ecological systems and raising public health concerns. The application of antibiotics to biofilms is usually ineffective because of their limited penetration into the biofilms or, worse, their stimulating the development of further antibiotic resistance by the biofilm-associated cells. All of these have contributed to the widely acknowledged challenges in biofilm inactivation and, therefore, generated great interest in the exploration of new and more effective alternative antimicrobial agents and strategies for the prevention of biofilm formation and eradication of biofilms. In the work reported here, the newly developed carbon “quantum” dots or carbon dots (CDots) (Sun et al., 2006; Sun, 2020), coupled with visible light are explored with significant success in both prevention and eradication efforts.

CDots are small carbon nanoparticles (CNPs) (diameter <10 nm) with surface passivation for which the most effective has been chemical functionalization with organic molecules (Sun, 2020). Their optical properties and unique photoexcited state redox processes have been investigated for a wide range of promising applications. In particular, the broad optical absorptions of CDots over the visible spectrum, extending into both near-UV and near-IR, are readily coupled with visible/natural light sources for photoinduced activities and functions, including those that are highly effective against various model bacteria, multidrug-resistant pathogens, and viruses (Dong et al., 2020a). For example, CDots with the simple diamine 2,2′-(ethylenedioxy)bis (ethylamine) (EDA) for surface functionalization, denoted as EDA-CDots, have been developed and validated as a benchmark for their well-characterized dot structures, electronic transitions, and photoexcited state properties and processes (LeCroy et al., 2014). Especially relevant to the purpose of the work reported here, EDA-CDots are shown to exhibit potent antibacterial and antiviral activities, effectively inactivating *E. coli, Bacillus subtilis*, and norovirus virus-like particles (VLPs) (Meziani et al., 2016; Al Awak et al., 2017; Dong et al., 2017a; Dong et al., 2018; Dong et al., 2020a). The objective of this study is to evaluate the light-activated antimicrobial functions of EDA-CDots in inhibiting the formation of biofilms and inactivating biofilm-associated bacterial cells. In the investigation, biofilms of *B. subtilis* were used as a model for both the inhibition of their formation and the inactivation of *B. subtilis* cells in those already formed.

## Materials and Methods

CDots. EDA passivated CDots, denoted as EDA-CDots, were synthesized as reported in our previous studies (LeCroy et al., 2014; Dong et al., 2017a; Dong et al., 2017b). Briefly, carbon nano-powder (2 g) (US Research Nanomaterials, Inc.) was refluxed in aqueous nitric acid (8 M, 200 ml) (VWR) for 48 h. The reaction mixture was cooled back to room temperature, followed by a centrifugation step at 1000 xg to remove the acid solution. The residue was dispersed in deionized (DI) H_2_O and dialyzed in a membrane tubing against freshwater for 48 h with a molecular weight cutoff of ∼500. The sample was centrifuged at 1000 xg to retain the supernatant. CNPs were recovered by the removal of water and then refluxed in neat thionyl chloride (Alfa Aesar) for 12 h. Thionyl chloride was then removed, the sample was mixed with dried EDA (Sigma-Aldrich) liquid in a round-bottom flask, heated to 120°C, and stirred vigorously under nitrogen protection for 3 days. After cooling down to room temperature, the reaction mix was dispersed in DI-H_2_O and then centrifuged at 20,000 xg to retain the supernatant. EDA-CDot solution was obtained by dialyzing supernatant in the membrane tubing (cutoff molecular weight ∼500) against freshwater to remove the unreacted EDA and other small species. Characterization by using NMR, microscopy, and optical spectroscopy techniques confirmed the structure and properties of EDA-CDots as previously reported (LeCroy et al., 2014).

Bacterial culture and cell preparation. Overnight, freshly grown *B. subtilis* cells in Luria-Bertani (LB) broth were harvested by centrifugation and then washed twice with PBS. The cells were resuspended in PBS or growth medium, and further dilutions with desired cell concentrations were prepared for experimental uses. The actual cell concentrations in the samples were determined by the traditional surface-plating method, in which the cell samples were 1/10 serial diluted, and aliquots of 100 µL of appropriate dilutions were plated on LB agar plates. The colonies were counted after 18 h incubation at 37°C, and calculated into colony-forming units (CFUs) per mL for the cell concentration in each sample.

Biofilm formation in the presence of CDots and with different CDots adding time. Overnight-grown *B. subtilis* bacterial cells in LB broth (15 ml) were washed once with PBS and then suspended in 10 ml tryptic soy broth (TSB). To test the effects of the presence of CDots and its adding time on the biofilm formation, *B. subtilis* cells were prepared as above. Aliquots of 150 µL 1/100 cells dilution in ½ TSB were added into the wells of a 96-well plate, followed by adding CDots (small volume) to reach the final concentration of 10, 20, or 30 μg/ml at 0 (initial time), 1, 2, 3, 4, 5, 6, 18, and 24 h during biofilm growth. Control samples without CDots were also included. Each sample was prepared in triplicate. The plates were incubated under visible light from a commercially acquired (The Home Depot) household A19 white-light LED bulb made by CREE (815 Lumens, 60 W incandescent light bulb equivalent according to the manufacturer’s specification), which was placed at ∼10 cm above the plates for 48 h in a 37°C incubator for biofilm development.

Measurement of biofilm formation and calculation of inhibitory effect of CDots on biofilm formation. After 48 h of biofilm formation, the formed biofilms were measured using the crystal violet staining method. Briefly, the cell suspensions in the wells were discarded, and the wells were washed with 200 µL sterile tap water once to remove the unattached cells. The plate was air-dried for 30 min, followed by staining with 180 µL of 0.2% crystal violet solution for 40 min at room temperature. The stain was discarded, and the wells were washed three times with 200 µL sterile tap water. The plate was air-dried for 20 min, and 200 µL of 30% acetic acid was added to the wells to resolve the stain. After sitting on the plate for 15 min at room temperature, the solution was gently mixed and the optical density (OD) at 550 nm wavelength was measured using the Max M5 spectrophotometer. The inhibitory effect of CDots on biofilm formation was calculated using the following formula:

$$Inhibitory\;Rate\left(\%\right)=\frac{OD_{550}\;of\;control\;sample-OD_{550}\;of\;CDots\;treated\;sample}{OD_{550}\;of\;control\;sample}\times 100\%$$

The dose response data was also analyzed using Prism 9 (GraphPad Software, LLC, San Diego, CA) nonlinear fitting to obtain the adding time at which 50% of the inhibitory effect was reached for different concentrations of CDots.

CDot treatment to planktonic *versus* biofilm-associated *B. subtilis* cells. For planktonic cell tests, overnight-grown *B. subtilis* cells in LB broth were harvested by centrifugation and then washed twice with PBS. The cells were resuspended in PBS and treated with CDots at various concentrations with the final volume of 150 µL in 96-well plates. The plates were placed on a shaker (Lab-Line Instruments, Inc., IL) at the setting of two under the illumination of visible light (the same setting as described above) for 1 h at room temperature. The control treatment in dark were also included as a comparison to the light treatments, for which the samples contained the same amount to cells and CDots, but the plates were wrapped with aluminum foil to be protected from light illumination. After the treatments, the samples were 1/10 serial diluted and plated on LB agar plates. Bacterial colonies were counted after 18 h incubation at 37°C for calculating the CFU of viable cells in each sample.

For biofilm-associated cell tests, mature biofilms were developed from *B. subtilis* cells in 200 µL of ½ TSB in 1.5 ml centrifuge tubes for 2 days. The solutions in the tubes were removed, and the tubes were rinsed with PBS. The biofilms were treated with CDots at the concentration of 10, 20, 30, and 40 μg/ml for 3 h under the illumination of visible light the same as described above, and control samples without CDots were included. After treatments, the CDot solutions were removed, and 200 µL PBS was added. The biofilms were detached and homogenized using a Branson 3,510 ultrasonic cleaner (Danbury, CT, United States) for 15 s and then vigorously vortexed for 2 min. The samples were serial diluted and plated on LB agar plates. The colonies were counted after 18 h incubation at 37°C and calculated into CFU/mL for the viable cell numbers in each sample.

CDots coupled with a chelating agent to treat *B. subtilis* biofilm. Mature *B. subtilis* biofilms were grown in 150 µL ½ TSB in 1.5 ml centrifuge tubes for 2 days. After the growth, the supernatants in the tubes were removed, and the biofilms were washed once with DI-H_2_O and used as the initial biofilm for treatments. The chelating agent, ethylenediaminetetraacetic acid (EDTA), was selected to use in the experiments. The treatment with a chelating agent on the biofilms was conducted at 37°C for 21 h by adding 200 µL Na_2_-EDTA solution in ½ TSB at various concentrations ranging from 0.5 to 5 mM into the tubes. After the treatment, the Na_2_-EDTA solution was removed, and 200 µL CDots solution at various concentrations ranging from 10 to 30 μg/ml was added for 1 h treatment under the illumination of visible light. After the removal of CDot solutions, the biofilms were washed once with DI-H_2_O. To enumerate the viable cells in the treated biofilms, the biofilms were detached from the well of the tube by adding 1 ml PBS solution to each tube, followed by sonication for 10 s using Bransonic^®^ ultrasonic cleaner 3,510 (Branson Ultrasonics Corporation, Danbury, CT, United States), and then by vortexing vigorously for 2 min. The biofilm cell suspension was serially diluted in PBS, and the appropriate dilutions were surface plated on TSA plates to determine the CFU/mL in each sample.

Statistical analyses. The test results were statistically analyzed using the SAS System 9.2 (SAS Institute Inc, Cary, NC, United States) with the general linear model (GLM), with *p* < 0.05 being considered as significantly different.

## Results and Discussion

### EDA-CDots

**FIGURE 1:**
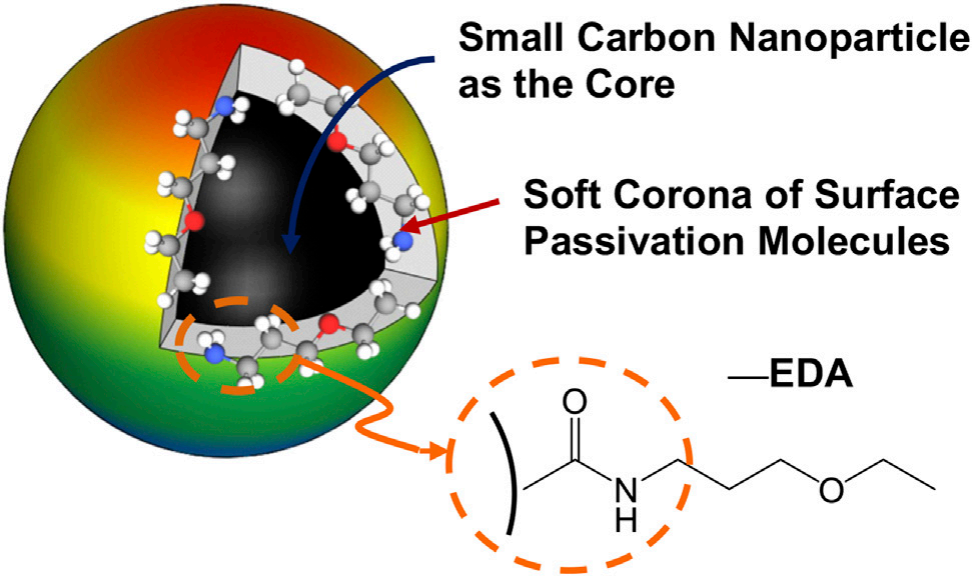
A cartoon illustration of an EDA-CDot, which is generally a small CNP core with attached and strongly adsorbed surface passivation molecules (a configuration similar to a soft corona).

**FIGURE 2:**
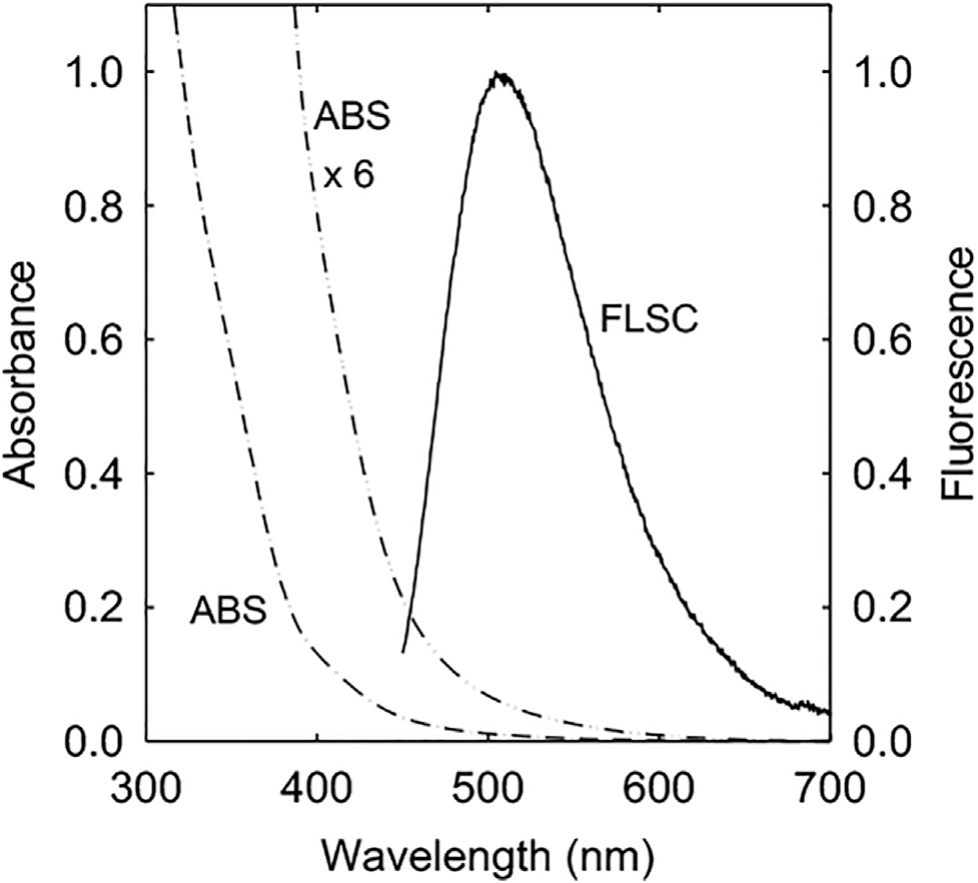
Absorption (ABS) and fluorescence (FLSC, 440 nm excitation) spectra of EDA-CDots in aqueous solution.

EDA-CDots were synthesized by chemical functionalization of the preprocessed and selected small CNPs with EDA molecules in amidation reactions (LeCroy et al., 2014; Dong et al., 2017a; Dong et al., 2017b). The dot sample was characterized by using NMR, microscopy, and optical spectroscopy techniques to have the results match those reported previously (LeCroy et al., 2014). The structure of EDA-CDots is illustrated in Figure 1. The CDots with the organic functionalities on the surface of small CNPs are more like dendritic polymers, thus readily soluble in water, with the resulting aqueous solution strongly absorptive in the visible spectrum (Figure 2). Their observed bright fluorescence emissions (Figure 2) suggest photoexcited state characteristics more favorable to the desired antibacterial function as established in the previous experimental correlations (Al Awak et al., 2017). The EDA-CDots in aqueous solution were used in the anti-biofilm experiments.

### Inhibitory Effect of CDots on *B. subtilis* Biofilm Formation

**FIGURE 3:**
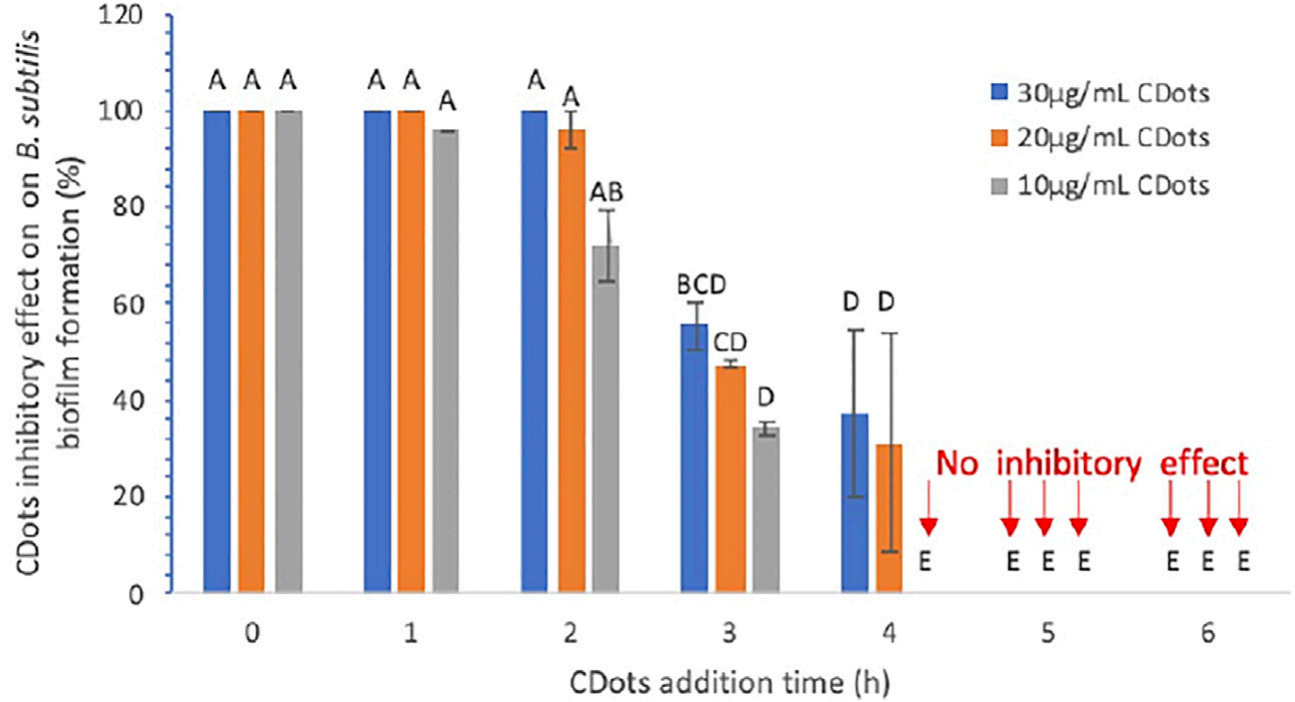
Inhibitory effect (in percentage) of CDots and their adding time on *B. subtilis* biofilm formation. Data is presented as the mean values with ±SD as error bars. Different letters above the columns indicate statistically significant differences (*p* < 0.05).

First, biofilm formation by *B. subtilis* in the presence of EDA-CDots under visible light illumination was evaluated. Experimentally, freshly overnight-grown *B. subtilis* cells were added into the wells of a 96-well plate, and EDA-CDot solutions were added and mixed at the final concentration of 10, 20, or 30 μg/ml with the total volume of 150 µL in ½ TSB. The plates were incubated under the illumination of visible light from a commercially acquired (The Home Depot) household A19 white-light LED bulb made by CREE (815 Lumens, 60 W incandescent light bulb equivalent according to the manufacturer’s specification) for 48 h in a 37°C incubator for the biofilm development. The light intensity at the plate surface was ∼4.8 mW/cm^2^. The biofilm formation after 48 h was assessed by using the crystal violet staining method. The inhibitory effect on the biofilm formation by CDots was quantified using the formula provided in Methods and Materials. The results indicated that, when 10, 20, or 30 μg/ml CDots were added at the very beginning (time 0) during biofilm growth, they were highly effective, completely inhibiting the biofilm formation at 48 h (Figure 3).

Furthermore, as also shown in Figure 3, the time of CDot addition during biofilm growth had a significant influence on the extent of the final biofilm formation. When the 10 μg/ml CDots were added at 1, 2, 3, and 4 h after the initiation of biofilm growth, the inhibitory effect on the final biofilm formation was decreased to 95.86, 72.20, 34.25, and 0%, respectively. The results also show that, even with a higher CDot concentration of 20 or 30 μg/ml, the CDots must be added within the first 2 h after the initiation of biofilm growth to achieve 100% or nearly 100% inhibitory effect on the final biofilm formation detected at 48 h. For all three tested CDot concentrations, when the CDots were added at 4 or 5 h after the initiation of biofilm growth, no inhibitory effect was observed. The analysis of dose-response data using a Prism nine nonlinear fitting dose-response model indicates that the CDot adding times to achieve 50% inhibition of biofilm formation by 10, 20, and 30 μg/ml CDots was 2.54, 3.1, and 3.32 h, respectively, under the given testing condition. Overall, the inhibitory efficacy of CDots on biofilm formation is dependent significantly on the time point when CDots were added; the earlier the CDots were added, the better inhibitory effect on the biofilm formation.

These results are understandable and explainable by considering the interactions between CDots and bacterial cells during the biofilm formation. At the early stage during biofilm formation, no thick EPS is produced around the bacteria, and most of the bacterial cells are still planktonic so that the added CDots could bind and interact with bacteria efficiently to inactivate the cells before they can form a biofilm, thus the observed high inhibitory effects on biofilm formation. If CDots are added 4–5 h after the initiation of biofilm growth, bacterial cells are multiplied, and the ECM is gradually fortified by the components of EPS with the growth of biofilm. During the growth, the formation of an ECM network may hinder the penetration of CDots into the biofilm and prevent the direct contact and interactions of CDots with the bacterial cells. Such contact and interactions are particularly relevant to the antimicrobial mechanism of light-activated CDots. As rationalized in terms of consistency with abundant experimental observations, in CDots upon photoexcitation, there must be rapid charge transfers and separation for the formation of electrons and holes, which are trapped at various stabilized surface defect sites. These separated redox pairs are credited for their major contributions to the observed antimicrobial activities (Dong et al., 2020b), mostly in the near-neighbor mode due to the short-lived nature of these redox species. Their radiative recombinations result in emissive excited states responsible for the observed bright and colorful fluorescence emissions and also the generation of classic reactive oxygen species (ROS), which also contribute to the antimicrobial function. The ROS are still short-lived, and their antibacterial activities may also be hindered by poor diffusion conditions associated with the ECM network during the biofilm formation. Thus, CDots with light activation are more effective in preventing biofilm formation before the bacterial cells have the opportunity and time to form the network structure toward the biofilm and less effective when the biofilm formation is already well on the way due to the limitation associated with the requirement for the CDots to penetrate into the biofilm. Such a limitation became more evident in evaluation on using EDA-CDots coupled with the same visible light exposure to eradicate mature biofilms.

### Photoexcited CDots for Inactivation of Planktonic *Versus* Biofilm-Associated Cells

**FIGURE 4:**
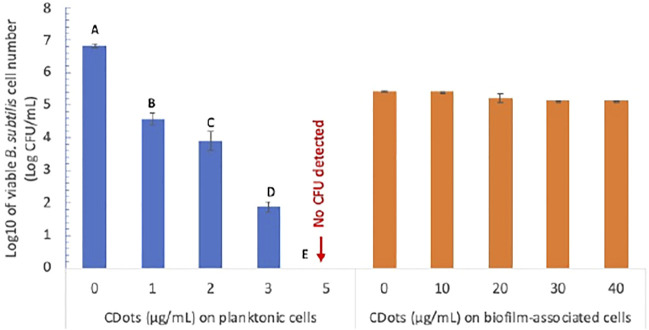
The antimicrobial effects of EDA-CDots on *B. subtilis* cells in planktonic and biofilm-associated origins with 1 h visible light treatment. Data is presented as the mean values with ±standard deviation as error bars. Statistical analysis was performed within the data of planktonic cells and within biofilm-associated cells, respectively. Different letters above the columns indicate statistically significant differences with *p* < 0.05; no statistical difference within the data of biofilm-associated cells.

The antibacterial action of light-activated EDA-CDots against planktonic *B. subtilis* cells *versus* the cells in biofilm was assessed experimentally. Figure 4 shows the viable cell numbers of *B. subtilis* after the planktonic cells were treated with EDA-CDots at concentrations of 1, 2, 3, and 5 μg/ml for 1 h under visible light along with the control samples without CDots but subject to otherwise the same treatment conditions. The CDot treatment at 1 μg/ml under visible light led to a significant reduction (*p* < 0.05) in viable cell numbers with more than 2 log reduction from 6.83 to 4.58 log. At a higher CDot concentration of 5 μg/ml, the 1 h treatment under visible light killed all the bacterial cells, achieving 6.83 log reduction in viable cells. The analysis of dose-response data using Prism nine nonlinear fitting indicated that the CDot concentration needed to achieve 50% log reduction of *B. subtilis* at the given treatment condition was 2.02 μg/ml. These results reaffirm the conclusion from previous observations that EDA-CDots are highly effective and efficient visible light-activated antibacterial agents toward planktonic *B. subtilis* cells (Meziani et al., 2016; Al Awak et al., 2017). However, the inactivation of the biofilm-associated *B. subtilis* cells was apparently much more difficult even with higher EDA-CDot concentrations coupled with longer light exposure.

Experimentally, *B. subtilis* biofilms were grown for 2 days. The planktonic bacteria in the growth medium was removed first, and the formed biofilms were rinsed with DI water to remove unattached cells. Then, EDA-CDots at higher concentrations than those for planktonic cells were added to treat the biofilms with light exposure time as long as 3 h. As also shown in Figure 4, the results on the viability of the cells in the biofilms after their treatment with EDA-CDots at concentrations of 10, 20, 30, and 40 μg/ml suggest only minor effects on the cells with percentage reductions in viable cell numbers of 0, 8.60, 34.65, and 34.06%, respectively. A conclusion from the comparison in Figure 4 is that the antibacterial efficacy of light-activated CDots on biofilm-associated cells is significantly lower than that on their planktonic counterparts.

The finding and conclusion above are consistent with those from other studies in which the biofilm-associated cells were found to be much more resistant to various antibacterial agents (Mah and O'Toole, 2001; Bridier et al., 2015). For instance, LeChevallier et al. reported that biofilm-associated bacteria grown on the surfaces of granular activated carbon particles, metal coupons, or glass microscope slides were 150 to more than 3,000 times more resistant to hypochlorous acid (free chlorine, pH 7.0) than planktonic cells, and the resistance of biofilm-associated bacteria to monochloramine disinfection was found to be two- to 100-fold higher than that of planktonic bacteria (Lechevallier et al., 1988). It is also shown that more than 99% planktonic *K. pneumonia* and HPC strain two could be inactivated by 24 h treatment with cupric sulfate (1 mg/L as copper) or sodium chlorite (5 mg/L), yet 1 mg/L copper or 10 mg/L sodium chlorite with the same treatment conditions had little antimicrobial effect on the biofilm-associated bacteria (Lechevallier et al., 1988).

Mechanistically, the EPS in biofilms is known to provide protection to the cells by blocking the access of antibiotics/antimicrobial reagents into the biofilms (Mah and O'Toole, 2001; Bridier et al., 2015), and the same protection might have impeded the penetration of CDots to reach the *B. subtilis* cells in biofilm. Among many factors and parameters that may affect drug/agent penetration properties, molecular weight, charge, and hydrophilic-hydrophobic balance are considered. On EDA-CDots, their sizes are larger than many molecular agents, but their close to spherical shape could be beneficial to the penetration. A more significant parameter for consideration might be the surface functionality of EDA-CDots as the abundant amino moieties become positively charged at near neutral pH, which is good for interactions with negatively charged bacterial cells but not so much for breaking though the blockage of EPS in biofilms. This may argue for modifications in the functional groups of CDots to manipulate the dot surface characteristics specifically for the penetration though the tasks are challenging and resource intensive. As an initial step in this work, an alternative approach was pursued by using agents that are capable of assisting EDA-CDots to assess and reach the biofilm-associated *B. subtilis* cells for the inactivation, yielding promising results.

### CDots Coupled With Chelating Agent for Photoinduced Inactivation of Biofilm-Associated Cells

**FIGURE 5:**
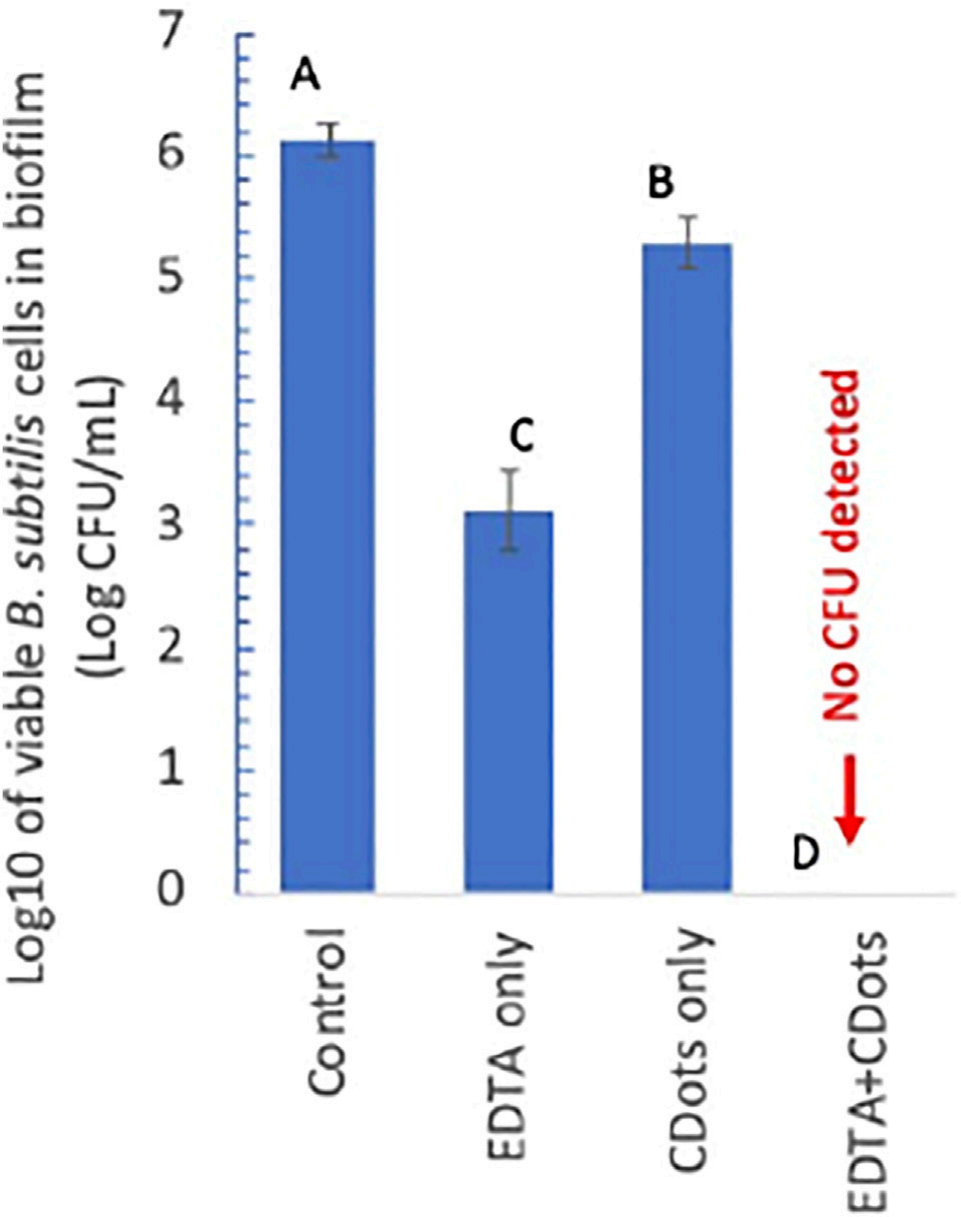
log10 of viable cell numbers in the biofilm-associated *B. subtilis* cell suspensions treated with 5 mM EDTA alone, 30 μg/mL CDots alone, and the combination of EDTA and CDots along with the untreated control. Different letters above the columns indicate statistically significant differences with *p* < 0.05.

The approach takes advantage of the growing efforts in the research field on strategies that specifically target the biofilm architecture to break or weaken the EPS protection of the biofilm-associated cells (Fleming and Rumbaugh, 2017). One strategy is to use chelating agents to scavenge metal cations that are critical to the EPS structure (Chaudhary and Payasi, 2012; Fleming and Rumbaugh, 2017). In this study, the well-established molecular chelator EDTA was identified and employed as co-anti-biofilm agent for the combination with EDA-CDots to inactivate the biofilm-associated *B. subtilis* cells. Experimentally, the same *B. subtilis* biofilms as those described above were created, and the biofilms were treated with solutions of EDTA sodium salt in ½ TSB (200 µL) of various final concentrations (0.5, 1, 2.5, and 5 mM) at 37°C for 21 h, followed by the treatment with EDA-CDots in various concentrations (200 μL, 10, 20, and 30 μg/ml) under visible light at ambient temperature for 1 h. The treated biofilms were washed with DI-H_2_O, and then PBS (1 ml) was added. The biofilms were detached by sonication for 10 s and then by vortexing vigorously for 2 min. The viable cell numbers in the detached-cell suspensions were determined by surface plating appropriate serial dilutions of the cell suspension on TSA plates and colony counting after 24 h incubation. The results show that the combination of EDTA and EDA-CDots/visible light treatments was highly effective for the inactivation of biofilm-associated *B. subtilis* cells with more than six log viable cell reduction by the combination of 5 mM EDTA and 30 μg/ml EDA-CDots (Figure 5). The results demonstrate that a known chelating agent such as EDTA could assist CDots to access and reach the *B. subtilis* cells in biofilm to achieve excellent inactivation outcomes. Mechanistically, the assistance is likely associated with the ability of EDTA molecules to extract metal cations that are critical to the stability of the EPS structure though more details on the related biofilm structural changes that allow the penetration of EDA-CDots to act on the biofilm-associated *B. subtilis* cells remain to be explored and understood. Nevertheless, the combination treatment represents a promising approach to realize the potent antibacterial function of light-activated CDots in the control and eradication of mature biofilms. Further exploration of the approach to evaluate other agents that may break or weaken the EPS protection to assist light-activated CDots in the inactivation of biofilm-associated bacteria may prove rewarding for both mechanistic elucidation and technological applications.

## Conclusion

In conclusion, CDots with visible light activation are highly effective in preventing biofilm formation, for which the best outcome is with the CDots added at the very early stage of the biofilm growth. The effective early intervention could be achieved with CDots at low concentrations. Thus, the treatment of CDots with visible light represents a viable preventative strategy for applications in which the formation of bacterial biofilms is a major problem. The destruction and eradication of mature biofilms are understandably more difficult due to the EPS fortification for which the EDA-CDots used in this study are apparently not enough by themselves. The promising outcomes with the combination of the CDots and a known chelating agent under visible light in their inactivation of the biofilm-associated bacterial cells have provided the initial validation on the strategy of equipping CDots, either on the dots or in mixtures with additional weapons designed to break the EPS defense of mature biofilms for the realization of the potent antimicrobial function of CDots. Further development and validation of such a strategy, including the evaluation of other agents for assisting the weakening and breaking of biofilm structures and also biofilms of other bacterial species, will be pursued in follow-on investigations.

## Data Availability

The original contributions presented in the study are included in the article/Supplementary Material, further inquiries can be directed to the corresponding authors.
